# Validation of reference genes as internal control for studying viral infections in cereals by quantitative real-time RT-PCR

**DOI:** 10.1186/1471-2229-10-146

**Published:** 2010-07-15

**Authors:** Jana Jarošová, Jiban K Kundu

**Affiliations:** 1 Department of Virology, Crop Research Institute, Drnovská 507, Prague - Ruzyně, the Czech Republic

## Abstract

**Background:**

Reference genes are commonly used as the endogenous normalisation measure for the relative quantification of target genes. The appropriate application of quantitative real-time PCR (RT-qPCR), however, requires the use of reference genes whose level of expression is not affected by the test, by general physiological conditions or by inter-individual variability. For this purpose, seven reference genes were investigated in tissues of the most important cereals (wheat, barley and oats). Titre of *Barley yellow dwarf virus *(BYDV) was determined in oats using relative quantification with different reference genes and absolute quantification, and the results were compared.

**Results:**

The expression of seven potential reference genes was evaluated in tissues of 180 healthy, physiologically stressed and virus-infected cereal plants. These genes were tested by RT-qPCR and ranked according to the stability of their expression using three different methods (two-way ANOVA, GeNorm and NormFinder tools). In most cases, the expression of all genes did not depend on abiotic stress conditions or virus infections. All the genes showed significant differences in expression among plant species. Glyceraldehyde-3-phosphate dehydrogenase (GAPDH), beta-tubulin (TUBB) and 18S ribosomal RNA (18S rRNA) always ranked as the three most stable genes. On the other hand, elongation factor-1 alpha (EF1A), eukaryotic initiation factor 4a (EIF4A), and 28S ribosomal RNA (28S rRNA) for barley and oat samples; and alpha-tubulin (TUBA) for wheat samples were consistently ranked as the less reliable controls.

The BYDV titre was determined in two oat varieties by RT-qPCR using three different quantification approaches. There were no significant differences between the absolute and relative quantifications, or between quantification using GAPDH + TUBB + TUBA +18S rRNA and EF1A + EIF4A + 28S rRNA. However, there were discrepancies between the results of individual assays.

**Conclusions:**

The geometric average of GAPDH, 18S rRNA and TUBB is suitable for normalisation of BYDV quantification in barley tissues. For wheat and oat samples, a combination of four genes is necessary: GAPDH, 18S rRNA, TUBB and EIF4A for wheat; and GAPDH, 18S rRNA, TUBB and TUBA for oat is recommended.

## Background

*Barley yellow dwarf virus *(BYDV) is one of the economically most important viral diseases of cereals worldwide. It can cause significant yield losses in major cereal crops like wheat, barley, rice, maize, oat and ryegrass [1]. Virtually all species of the family Poaceae (Graminae) can be infected, providing > 150 species as putative sources of these viruses [2].

In many cases, the virus titre in plants and its ability to multiply need not correspond with symptomatic manifestation of the infection. For example, this can be the case with tolerant plants [3], plants with high nitrogen uptake [4], and can also be dependent on environment conditions [5]. Furthermore, it is often a combination of different viral genes' expression that determines the severity of symptoms [6]. Quantification by quantitative real-time PCR (RT-qPCR) makes it possible to monitor the titre of all the different viral genes as well as to quantify the virus particles. The determination of individual viral genes' expression can greatly enhance our knowledge and reveal many aspects of disease aetiology and virus ecology.

However, for such methods, normalisation is required to correct for any variation in RNA integrity, reverse transcription efficiency, and initial sample amount among different samples [7]. Different approaches have been proposed to normalise measurements of expression levels [8], but this is generally done using an internal 'reference gene', under the assumption that this has a constant level of expression in the chosen tissue, is not affected by the treatment and has no inter-individual variability [9]. Therefore, the most prominent problem in quantitative RNA expression analysis is the selection of an appropriate reference gene. Misinterpretation of data occurs when expression measures are erroneously normalised to a subset of mRNAs that are subject to strong regulation [10,11]. While it seems unreasonable that the transcription of any gene in a living cell is absolutely resistant to cell cycle fluctuations or nutrient status, it is important to identify candidate genes that are at least minimally regulated during the individual experiment to allow the accuracy of RNA transcription analysis that real-time PCR offers. The correct reference genes can be selected by evaluating data from RT-qPCR with statistical algorithms such as GeNorm [12], BestKeeper [13] or NormFinder [14]. Commonly used reference genes for normalisation of RT-qPCR data in plants include ACTβ, TUBA, TUBB, EF-1-α, EIF4A, GAPDH, and 18S, 25S and 28S rRNAs [15-19].

In this paper, commonly used reference genes' expressions in wheat, barley and oats under abiotic and biotic [*Barley yellow dwarf virus *(BYDV) infection] stress were compared. Furthermore, an assay for BYDV relative quantification in these cereal species was developed. Different approaches of RT-qPCR were tested in a case study that quantified the titre of BYDV in tolerant and susceptible oat varieties.

## Results and discussion

**Figure 1:**
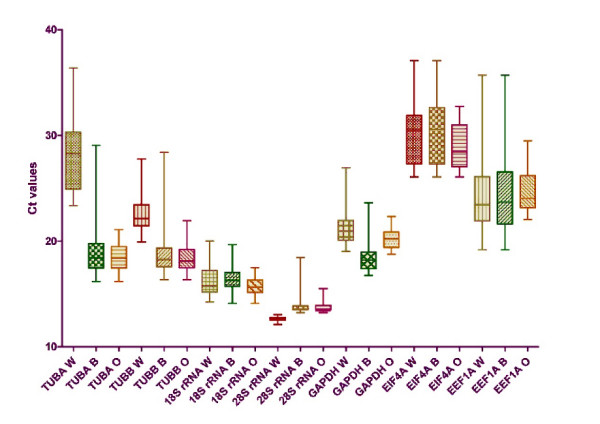
**C_q _values distribution of candidate reference genes in different species**. The values are given as real-time PCR quantification cycle (C_q_) values. The boxes represent the lower and upper quartiles with medians; the whiskers illustrate the 10-90 percentiles of the samples. All C_q _values significantly differed between species (one-way ANOVA). (W) = wheat samples (60 samples), (B) = barley samples (60 samples), (O) = oat samples (60 samples).

In this study, the stability of gene expression of genes normally used as reference genes in relative quantification by RT-qPCR was tested with the aim of developing an optimal and accurate assay for quantification of BYDV in leaf tissues of wheat, barley and oats. A hypothesis of finding reference genes suitable for inter-species testing (barley, wheat and oats) was also tested. The data obtained from experiments for each gene and the virus were analysed using three different methods. The relative gene expressions of individual genes were measured by RT-qPCR and compared by NormFinder [14] and GeNorm [12] tools. Also, the raw quantification cycle (C_q_) values (Fig. 1) of individual genes for specific species and treatments were analysed by two-way analysis of variance (ANOVA). In this way, to compare the different RNA transcription levels, the C_q _values were compared directly; whereas to ensure comparability among the RT-qPCR assays of the seven reference genes, we first determined the PCR efficiency of each individual assay by measuring serial dilutions of 100 ng cDNA from barley, wheat and oat samples, all in triplicate. Only C_q _values < 40 were used for the calculation of the PCR efficiency from the given slope software according to the equation: PCR efficiency = (10^[-1/slope] ^- 1) × 100. All PCRs had efficiencies of 85-101%. Intra-assay variation was < 1% and inter-assay variation < 4.5% for all assays.

Furthermore, the titre of BYDV in two oat varieties (BYDV susceptible and BYDV tolerant) was quantified by absolute quantification with cloned standards and no reference gene, and by relative quantification with the reference genes that performed best and worst in the NormFinder and GeNorm analyses.

### ANOVA results

Firstly, two-way ANOVA was used to compare the influence of species and stress factors on raw C_q _values of individual genes (Fig. 1). Probabilities of *P *> 0.05 were considered non significant, *P *< 0.01 as very significant and *P *< 0.001 as extremely significant. The analysis revealed an extremely strong influence of species (Table 1). The impact of stress factors of the plant (healthy, and abiotic and biotic stress) was not significant in cases of 28S rRNA, EIF4A, and EF1A; significant in cases of TUBA, TUBB and GAPDH; and very significant for 18S rRNA. However, for 18S rRNA, TUBA, TUBB, EIF4A and GAPDH, the interactions were also of some significance and therefore the results should be interpreted carefully. However, the influence of the species was very clear. This was the first indication that the inter-species comparison hypothesis may be rejected because the tested genes differed in expression among the species.

**Table 1:** Two-way ANOVA results

Gene	Fitness	Species	Interaction
28S rRNA	ns	***	ns
18S rRNA	**	***	**
TUBA	*	***	**
TUBB	*	***	**
EIF4A	ns	***	*
EF1A	ns	***	ns
GAPDH	*	***	**

P > 0.05 considered as non significant (ns), P < 0.01 as significant (*), P < 0.001 as very significant (**), and P < 0.0001 as extremely significant (***).

Thereafter, a Bonferroni post-test was applied to the data to compare the value of each column (factor 'fitness') and each row (factor 'species') (Table 2). As expected, effects of 28S rRNA, EIF4A and EEF1A were not significant. There were significant differences for wheat samples among all three groups for TUBA and GAPDH; and between virus-infected samples and the other two groups for TUBB. In the case of 18S rRNA, the C_q _values of samples suffering from abiotic stress differed from the other stresses for all three species. The impact of species on the raw C_q _values seemed extremely relevant, and thus expression stabilities of the reference genes were assessed for three independent sets (wheat, barley and oats) as well as combined.

**Table 2:** Results of Bonferroni tests

Gene	Healthy vs. Abiotic stress			Healthy vs. Virus Infected			Abiotic stress vs. Virus infected		
	barley	oat	wheat	barley	oat	wheat	barley	oat	wheat
28S rRNA	ns	ns	ns	ns	ns	ns	ns	ns	ns
18S rRNA	***	***	***	***	ns	ns	ns	*	***
TUBA	ns	ns	***	ns	ns	**	ns	ns	***
TUBB	ns	ns	ns	ns	ns	**	ns	ns	***
EIF4A	ns	ns	ns	ns	ns	ns	ns	ns	ns
EF1A	ns	ns	ns	ns	ns	ns	ns	ns	ns
GAPDH	ns	ns	***	ns	ns	***	ns	ns	***

Results of Bonferroni tests applied on the data to compare the value of each column (factor 'fitness' - healthy; biotic stress, or abiotic stress) and each row (factor 'species').

P > 0.05 considered as non significant (ns), P < 0.01 as significant (*), P < 0.001 as very significant (**), and P < 0.0001 as extremely significant (***).

### GeNorm Results

**Figure 2:**
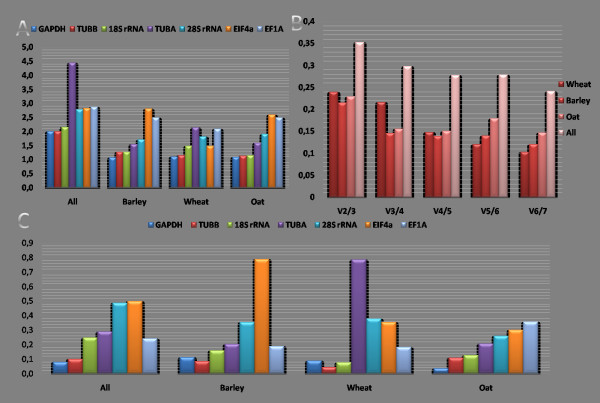
**Stability indices (A), and pairwise variation to determine the optimal number of normalisation genes (B) calculated with GeNorm, and stability indices calculated with NormFinder (C)**. (A) and (C): The stability indices are shown for all species combined, and also barley, wheat and oats individually. The stability of gene expression is inversely proportional to the stability index, so least stable genes have the lowest achieved values and vice versa. (B): The optimal number of genes was determined separately for barley, wheat and oats as well as for all samples combined (all). The recommended cut-off value under which there is no need for inclusion of another gene is 0.15.

GeNorm v3.4 software was used to analyse the expression stability of tested genes in various samples, and to rank them accordingly. The GeNorm is a statistical algorithm which determines the gene stability measure (M) of all the investigated genes, based on the geometric averaging of multiple reference genes and mean pairwise variation of a gene from all other reference genes in a given set of samples [12]. It relies on the principle that the expression ratio of two ideal reference genes is identical in all the samples, regardless of experimental condition and cell-type. Genes with the lowest M-values have the most stable expression. We analysed our data in two sets, one with all samples combined and the second according to plant species. When all the samples were combined, the average M-value of GAPDH and TUBB was lowest, and that of TUBA was highest (Table 3, Fig. 2A). The results remained very similar, when the M-value was measured for species series, with least value for GAPDH and TUBB (Table 3, Fig. 2A). According to the GeNorm tool, the TUBA was by far the least reliable reference gene when all samples were combined as one set, whereas for barley and oats the worst reference gene was EIF4A, and for wheat it was TUBA.

**Table 3:** Ranking of candidate reference genes and choice of best pair of reference genes by NormFinder and GeNorm tools

gene	GeNorm stability value (M)					NormFinder stability value			
	wheat	barley	oat	all	wheat	barley	oat	all	
GAPDH	1,09	1,06	1,08	2,0	0,088	0,112	0,035	0,074	
**TUBB**	1,14	1,27	1,12	2,0	0,041	0,085	0,108	0,098	
TUBA	2,13	1,54	1,59	4,43	0,785	0,201	0,205	0,289	
18S rRNA	1,48	1,27	1,14	2,15	0,076	0,158	0,125	0,248	
28S rRNA	1,82	1,71	1,90	2,78	0,378	0,354	0,258	0,487	
EIF4a	1,48	2,81	2,59	2,82	0,352	0,489	0,301	0,499	
EEF1A	2,08	2,48	2,49	2,87	0,181	0,187	0,355	0,241	

The greater expression of stability is indicated by a lower stability value (M). GeNorm stability is based on an estimate of the pairwise variation (M). For NormFinder analysis, samples were grouped into healthy, abiotic stress, and virus-infected groups. The stability is calculated from the intra- and inter-group variation and the best combination of genes is also given.

Theoretically, the high expression stability of a gene indicates that the use of a single reference gene is appropriate. However, for many studies, no single gene may be adequate and may require normalisation with two or more stable reference genes. Therefore, pairwise variations were calculated using GeNorm for each data set to determine the optimal number of reference genes for normalisation. For this, first the normalisation factors (NF) were calculated for the most stable reference genes (with lowest M-value) and then for other genes by stepwise inclusion of the reference gene that remains most stable. Subsequently, pairwise variations of NF_n _and NF_n+1 _were calculated, reflecting the effect of including additional (n + 1) gene.

For wheat and oats, four genes with the most stable expression were optimal for reliable normalisation with a pairwise variation cut-off value of 0.15, whereas for barley three genes were necessary (Fig. 2B). Below this cut-off value there is no need for inclusion of an additional reference gene. It was apparent that the inclusion of a fourth/fifth gene had no significant effect on pairwise variation. However, an attempt to calculate the optimal number of reference genes for all samples combined failed; even if all the reference genes were taken into account, the pairwise variation exceeded the cut-off value of 0.15 (Fig. 2B). The quantification might not be accurately normalised even if all the tested genes were used in the assay. This was additional evidence of the infeasibility of comparing BYDV between different cereal species by relative quantification, and also further confirmed the suspicion raised by analysis of raw C_q _values that the expression of the tested genes differed among species.

### NormFinder results

NormFinder is another approach to assess the stability of expression of reference genes [14]. In the present study, NormFinder indicated that the genes with the most stable levels of transcript abundance were GAPDH and TUBB for all the samples taken as one set and for barley and oat samples, and TUBB and 18S rRNA for wheat samples (Table 3, Fig. 2C). These were almost the same genes as those identified by GeNorm, with the exception that 18S rRNA performed better than GAPDH for wheat samples in NormFinder. The least stable genes, according to NormFinder, were EIF4A for all the samples analysed as one set and also for barley, TUBA for wheat samples, and EF1A for oat samples. Therefore, there was a very strong correlation between the results obtained from GeNorm and NormFinder, despite the fact that the methods of calculation are fundamentally different. NormFinder ranks reference genes according to the least estimated intra- and inter-group variation, which is more effective to control the influence of co-regulation of reference genes. NormFinder can account for heterogeneity in the tested samples, such as different treatment groups, and so distinguishes between stability and bias.

The wheat samples differed more from barley and oat samples than these two species differed from each other. This was especially the case for TUBA which performed well for barley and oats but very poorly for wheat. However, there were significant differences in expression of most of the genes, even between oats and barley, and therefore no intra-species comparison by real-time relative RT-qPCR can be recommended.

### Choice of reference genes

The results of NormFinder and GeNorm analyses corresponded well. The two tools have not always agreed on the particular order of the individual genes' expressions' stability values; however, the final choice of the best reference genes was almost uniform. The calculation of V-values by GeNorm for the proposed genes (Fig. 2B) is useful for deciding the optimal number to be used in an expression study; pairwise variation between samples is reduced by the inclusion of additional reference genes and therefore indicates the number of genes required to achieve an arbitrarily selected threshold of reference gene stability; a recommended cut-off value is 0.15. If we took into account the results of the pairwise variations, then four genes were necessary for wheat and oats, and three genes for barley. For barley, the three genes with the most stable expression across the samples as determined by GeNorm and NormFinder were GAPDH, 18S rRNA and TUBB. For wheat and oat, the combination of GAPDH, 18S rRNA, TUBB and TUBA (oat) or EIF4A (wheat) should provide safe normalisation. For oats and barley, the poorly performing genes were 28S rRNA, EIF4A and EF1A; these could not be recommended as reference genes for these species. The use of TUBA as a reference gene is not recommended as appropriate for wheat tissues, as with an M-value of 2.13 it appears to be regulated.

### Virus titre comparison results

**Figure 3:**
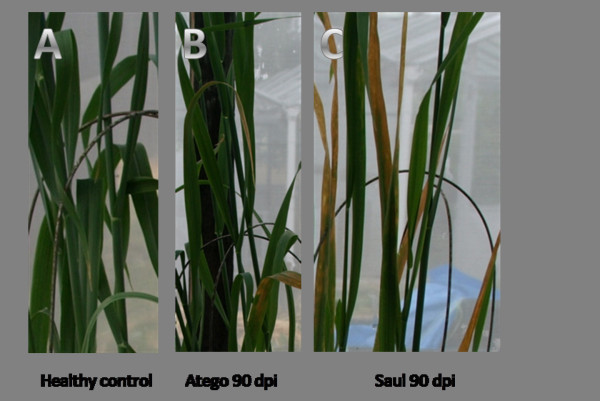
**BYDV symptoms manifest in two oat varieties compared to a healthy control plant**. On the left (A), is a healthy control; in the middle (B) the tolerant cv. Atego, and on the right (C), susceptible cv. Saul.

**Figure 4:**
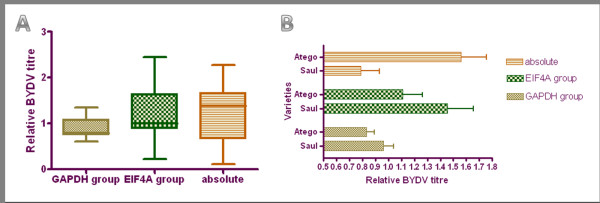
**Titre of BYDV in two oat varieties as determined by three quantification approaches**. (A): The relative BYDV titre levels in all oat samples determined by relative quantification using GAPDH, TUBB, TUBA and 18S rRNA (GAPDH group); 28S rRNA, EIF4A and EF1A (EIF4A group); and by absolute quantification with cloned standards (absolute). The absolute numbers were transformed into relative ratios for comparison. The boxes represent the lower and upper quartiles with medians; the whiskers illustrate the 10-90 percentiles of the samples. (B): The relative BYDV titre levels in the two oat cvs. Atego (tolerant) and Saul (susceptible) determined by relative quantification using GAPDH, TUBB, TUBA and 18S rRNA (GAPDH group); 28S rRNA, EIF4A and EF1A (EIF4A group); and by absolute quantification with cloned standards (absolute). The absolute numbers were transformed into relative ratios for comparison. No significant differences were recorded.

The analyses described above were applied to the quantification of BYDV in the oat samples. Oats were chosen because the differences in symptoms manifest between the susceptible and the tolerant variety were the greatest (Fig. 3). The virus titre was calculated under three different settings. First, the absolute quantification with cloned standards and no normalisation gene was carried out, as it is still a common practise of real-time quantification of viruses among plant virologists. Secondly, 28S rRNA, EIF4A and EF1A were chosen as reference genes that performed very poorly in both the GeNorm and NormFinder analyses; and finally, GAPDH, TUBB, TUBA and 18S rRNA were chosen as reference genes that performed best in both analyses. The results were analysed by one- and two-way ANOVA (Fig. 4).

There were no significant differences between the viral titre in cv. Saul (susceptible) and cv. Atego (tolerant) according to relative quantification, independently of the reference genes used in the assays. However, the trends of the two reference genes groups were slightly different (Fig. 4B). The GAPDH, TUBB, TUBA and 18S rRNA assays resulted in almost identical data from the two groups; however, the 28S rRNA, EIF4A and EF1A results might lead to the assumption that the tolerant cv. Atego had a lower virus load than the susceptible variety. Furthermore, the BYDV titre was determined to be statistically higher in the tolerant compared to the susceptible variety, by absolute quantification. This seems quite unlikely, and contrary to the plant symptoms, indicating that the absolute quantification with no normalisation is inadequate to quantify viruses in plants. These results support the necessity of the correct choice of reference genes for valid experimental data.

A similar study was conducted by Balaji et al. [20], in which the titre of BYDV-PAV was determined in susceptible and resistant wheat lines, using 18S rRNA as a reference gene. In their study, the titre of the virus was measured 1, 2, 4, 6, 8, 10, 12, and 14 d post-infection (dpi); the greatest differences were in the early stages of infection (4 dpi), when the titre varied as much as tenfold, the most susceptible wheat line being that with higher titre. However, later in infection (≥ 12 dpi), the titre in the susceptible line decreased to almost the same as in the resistant line. As the oat samples in this study were collected at 30 dpi, when the symptom differences appeared, the results of the relative quantification seem to be accordant. Nevertheless, for the comparison of such subtle differences, missing or inappropriately chosen normalisation could easily lead to erroneous interpretation of data.

## Conclusions

Our results indicate GAPDH, TUBB and 18S rRNA were the most stable reference genes for virus-infected cereals, giving very good statistical reliability according to the two software packages employed. Moreover, the use of four reference genes (GAPDH, 18S rRNA, TUBB and TUBA-oat/EIF4A-wheat) was sufficient for a reliable normalisation of the viral genes in wheat and oats, and three genes (GAPDH, TUBB and 18S rRNA) were necessary for barley. The absolute quantification with no reference gene is not recommended, since it might lead to inaccurate and misleading conclusions, due to lack of normalising factors. Studies aimed at the relative comparison of the BYDV titre among different cereal species also seem to be unfeasible because of different gene expression between species.

## Methods

### Plant, virus and vector material

Laboratory isolates of BYDV-PAV and BYDV-PAS maintained on barley seedlings and transmitted by aphids *Rhopalosiphum padi *and *Sitobion avenae *were used for the study. For each of wheat (*Triticum aestivum *L.), barley (*Hordeum vulgare *L.) and oats (*Avena sativa *L.), 60 plants were sown into individual pots. For each species, 30 plants were of the BYDV-susceptible and 30 of the BYDV-tolerant variety: wheat cvs. Anza (tolerant) and Vlada (susceptible); oat cvs. Atego (tolerant) and Saul (susceptible); and barley cvs. Wysor (tolerant) and Finesse (susceptible). They were maintained in greenhouses with controlled conditions of 16-h-light period and 20°C. When the plants were at the growth stage where two leaves were unfolded, 20 plants per species (10 per variety) were separated and infected with BYDV-PAS strain by vector transmission. For each plant, approximately five viruliferous *R. padi *aphids were transferred onto and allowed to feed on the plant for 2 d. After that, the plants were treated with insecticide and kept in the greenhouses for one more week. On the last day of the week period, 20 additional plants of each species were separated and abiotic stress conditions were created for them. The plants were moved into a chamber with 4°C constant temperature and darkness and were kept there for 24 h. Then, all 180 plants were harvested and the whole aboveground biomass of individual plants was used for further analysis.

For the case study, oat plants from a field experiment testing the resistance of different varieties to BYDV were used: cvs. Atego and Saul. Atego did not manifest many symptoms, while Saul symptomatically reacted as very sensitive to the infection. The leaf samples from 20 randomly chosen plants were collected one month after the inoculation with BYDV-PAV and BYDV-PAS. For each plant, the most symptomatic and the least symptomatic leaves were chosen.

### RNA purification and cDNA synthesis

The whole plants were ground with a mortar and a pestle in liquid nitrogen, and 100 mg of the material was then used for RNA isolation. The RNA was isolated with a Spectrum™ Plant Total RNA Kit (Sigma Aldrich) according to the manufacturer's instructions. The concentration and purity of the isolated RNA was then spectrophotometrically measured. Of total RNA, 5 μg was diluted to a total volume of 25 μL and digested with DNase I (DNA-*free*™; Ambion) according to the manufacturer's instructions. cDNA was produced using the M-MLV Reverse Transcriptase (Promega) according to the manufacturer's recommendations for oligo(dT)20 or random hexamers primed cDNA-synthesis. cDNA synthesis was performed using 1 μg of RNA, at 40°C. Finally, cDNA was diluted 1:5 before use in qPCR.

### Standards RNA preparation

A specific BYDV nucleotide sequence (294 bp) amplified by RT-PCR was inserted into the vector pGem-T (Promega) and cloned into *E. coli *JM-109. The plasmid was linearised at the *Rsa*I site and used as a target in an *in vitro *transcription reaction performed with Megascript T7 kit (Ambion) followed by DNase I (Ambion). The amount of RNA was quantified by spectrophotometry (Nanophotometer, Implen). The μg of single stranded RNA was converted to ρmol using the average molecular weight of a ribonucleotide (340 Da) and the number of bases of the transcript (Nb). The following mathematical formula was applied: ρmol of ssRNA = μg (of ssRNA) × (106 ρg/1 μg) × (1 ρmol/340 ρg) × (1/Nb). Avogadro's constant was used to estimate the number of transcripts (6.023 × 1023 molecules/mol). Thereafter, tenfold serial dilutions of the transcripts were prepared.

### Real-Time PCR conditions

Real-time PCR was performed using a 7300 Real-Time PCR system (Applied Biosystems). For analysis with SYBR Green I, PCR cycling consisted of three steps that included: 2 min incubation at 95°C followed by 40 cycles of 15 s at 95°C, and 60 s at 60°C; and finally the dissociation curve step of 15 s at 95°C, 60 s at 60°C, and 15 s at 95°C. Fluorescence readings were taken during the annealing/extension step (60°C incubation). The quantification cycle (C_q_) values for each reaction were calculated automatically by the 7300 Real-Time PCR system detection software by determining the point in time (PCR cycle number) at which the reporter fluorescence exceeded the computer-determined standard deviation for background by a factor of 10. The PCR Mastermix comprised of the primers (1 μL primer pair mix of 10 μM primer pair stock), 12.5 μL of 2 × Power Sybr Green Master mix (Applied Biosystems), and sterile nuclease free water to a final volume of 20 μL. Finally, 5 μL of cDNA was added to this mixture. The primers, genes and PCR conditions are listed in Table 4.

**Table 4:** Characteristics of gene specific real-time RT-PCR assays

*Gene symbol*	*Gene name*	*Accession No.*	*Primer sequence (5'→3')*	*Amplicon size*	*PCR efficiency*
**GAPDH**	Glyceraldehyde-3-phosphate dehydrogenase	AK251456	Forward: TGTCCATGCCATGACTGCAA	105	101%
			Reverse: CCAGTGCTGCTTGGAATGATG		
**TUBA**	Alpha tubulin-2B	AK250165	Forward: TTCGCCCGTGGTCATTACA	113	100%
			Reverse: GCATTGAAGACAAGGAAGCCC		
**TUBB**	Beta-tubulin	U76897	Forward: CAAGGAGGTGGACGAGCAGATG	84	97%
			Reverse: GACTTGACGTTGTTGGGGATCCA		
**ELF1A**	Elongation factor-1 alpha	AF479046	Forward: CAGTGCTGGACTGCCACA	164	91%
			Reverse: CTCCACCACCATGGGCTT		
**EIF4A**	Eukaryotic initiation factor 4a	EU850433	Forward: TTGTGCTGGATGAAGCTGATG	76	99%
			Reverse: ACACCAACAGCCACAGTTTGC		
**18S rRNA**	18S ribosomal RNA	M82356	Forward: GTGACGGGTGACGGAGAATT	151	85%, 99%*
			Reverse: GACACTAATGCGCCCGGTAT		
**28s rRNA**	28S ribosomal RNA	M82206	Forward: CCTGATCTTCTGTGAAGGGTTCGA	172	94%
			Reverse: GGTTCGATTAGTCTTTCGCCCCTA		
**BYDV cp**	coat protein of BYDV	EF521849	Reverse:TGTTGAGGAGTCTACCTATTTG	294	99%
			Forward:GTTGAGTTTAAGTCACACGC		

PCR efficiency was calculated as follows: efficiency = 10 ^(1/slope) ^- 1, expressed as a percentage.

* 85% is the efficiency of assays in which samples of wheat were analysed, 99% efficiency was recorded for barley and oat samples.

### Results analysis

The relative gene expression ratios were calculated by a mathematical model, which includes an efficiency correction for real-time PCR efficiency of the individual transcripts [8]. The amplification efficiency was established for each of the targets from serial dilutions of cereal leaves within range 0.80-1.0. The absolute viral gene's quantification values were transformed into relative values by simple proportion. Two-way ANOVA was performed on data, followed by the Bonferroni post-tests, where appropriate. Data in graphs or tables are presented as means with their standard errors of the means. *P *> 0.05 was considered as non-significant, *P *< 0.01 very significant and *P *< 0.001 extremely significant. Reference gene selective analysis was performed with the NormFinder [14] and GeNorm [12] tools.

## Authors' contributions

JJ performed all the experimental procedures and statistical calculations, carried out drafting and writing the manuscript, and prepared the figures. JKK conceived the project, provided guidance in the study design, made special data analysis, and helped in drafting the manuscript. Both authors were involved in revising the manuscript in progress, and critically reading and approving the final manuscript.
